# Childbirth in Bhutan: A study on the use of neuraxial analgesia for labor pain

**DOI:** 10.1002/puh2.73

**Published:** 2023-03-07

**Authors:** Tenzin Yoezer, Dawa Gyeltshen, Jampel Tshering

**Affiliations:** ^1^ Tsirang District Hospital Ministry of Health, Royal Government of Bhutan Tsirang Bhutan; ^2^ Department of Adult Intensive Care Unit Jigme Dorji Wangchuck National Referral Hospital Thimphu Bhutan; ^3^ Department of Anesthesiology Jigme Dorji Wangchuck National Referral Hospital Thimphu Bhutan

**Keywords:** anesthesia, Bhutan, childbirth, epidural analgesia, labor analgesia, labor pain, neuraxial analgesia, pregnancy

## Abstract

**Background:**

The practice of neuraxial labor analgesia (NLA) as a mode of pain relief was introduced in Bhutan in 2016 despite it being in practice for more than five decades. There is a lack of data on NLA in Bhutan. Therefore, this study describes the use of NLA and its outcome over 4 years in Bhutan.

**Methods:**

A retrospective descriptive study was conducted from 1 January 2018 to 31 December 2021. The data were obtained from the Anesthesia Department, medical records, admission forms, and birth‐registers. A total of 524 women were included. Data were recorded using 2021 Microsoft Excel version 16.57 (22011101) and analyzed using Epi Info 7.2.5.0. Categorical data were summarized using frequencies and percentages. Continuous data were summarized using mean and standard deviation.

**Results:**

The incidence of NLA usage was 3.5% (524/15,119). Most women were between 21 and 30 years (67.2%). Modes of delivery following NLA were spontaneous vaginal delivery, cesarean section, and assisted vaginal delivery 63.4%, 18.4%, and 18.3%, respectively. Non‐reassuring fetal status (37.5%) and failed progression of labor (36.5%) were the leading indications of cesarean section. The primary reason for assisted vaginal delivery was poor maternal effort (55.2%). Neonates of the mother who received neuraxial analgesia had neonatal jaundice in 8%, neonatal intensive care unit admission in 0.76%, and Apgar score less than 7 in 5.1% and 0.2% at 1 and 5‐min, respectively, after birth.

**Conclusion:**

Although NLA is safe for both mothers and babies, its use is low in Bhutan. The probable reason for the low incidence could be a shortage of anesthesiologists, cultural beliefs, and lack of awareness. The Ministry of Health and the Department of Anesthesia should work together to improve the service.

## INTRODUCTION

Giving birth is a joyful event celebrated by all cultures. However, childbirth and labor pain are traumatic experiences women go through in their life. Labor pain is comparable to phantom limb pain, postherpetic neuralgia, and cancer pain [1]. The American College of Obstetricians and Gynecologists (ACOG) [2] and the World Health Organization (WHO) [3] recommend offering epidural analgesia if women request pain relief unless there is a medical contraindication. Overall, 87% of women want their labor pain treated medically [4]. Every pregnant woman going through labor and childbirth has the right to ask for labor analgesia.

There are pharmacological and non‐pharmacological methods of pain relief. Lamaze, immersion in water, acupuncture, and massage are non‐pharmacological methods, whereas pharmacological options include neuraxial analgesia, nitrous oxide inhalation, systemic opioids, and local analgesia [5]. Among all forms of analgesia, neuraxial analgesia is the gold standard [5–7]. It is safe, effective, which has high maternal satisfaction compared to other pharmacological and non‐pharmacological methods [5, 6, 7]. Neuraxial labor analgesia (NLA) is a collective term for epidural, spinal, and combined spinal–epidural (CSE) analgesia.

However, NLA has the risk of accidental dural puncture, headache, hypotension, itchiness, fever, infection, and anaphylaxis [5, 6, 7]. It also carries a myth of increased cesarean rates, labor prolongation, instrumental deliveries, and future backache [5, 6, 8]. Previous studies have also found that NLA increases neonatal intensive care unit (NICU) admission, neonatal birth injury from instrumental delivery, and respiratory depression [5, 8]. However, Cochrane and systemic reviews showed that NLA did not increase caesarean rate, long‐term backache, and neonatal admission to NICU [5, 7].

NLA is common in the USA and Europe [9, 10] compared to the Asian countries [11, 12]. In developing countries, the data on women receiving labor analgesia are limited. However, in the west, NLA services have been available for over 50 years [13], and Bhutan just started offering them to mothers in 2016. Nonetheless, the service is available only in Jigme Dorji Wangchuck National Referral Hospital (JDWNRH), an apex hospital based in the capital, Thimphu. It is the largest hospital in the country with delivery of over 4000 babies in 2021 [14]. It is provided round the clock by the anesthesiologists and resident anesthesiologists under supervision. Currently in JDWNRH, there are two national anesthesiologists, four foreign anesthesiologists, and five residents. In addition to attending the routine operation theaters, they are also responsible for providing NLA. Women from other hospitals seeking the service come to the JDWNRH.

In 2016, the service was introduced as a gift from Her Majesty The Queen, Jetsun Pema. Earlier, mothers who wished to get NLA went abroad at their own expense. A shortage of anesthesiologists in the country had delayed the start. Although the NLA service has been available for over 5 years, studies have yet to assess its outcome on women and children. Without the local data on its outcomes, such as safety as the basis, it is possible that women have hesitated to avail the service. Therefore, this study aimed to find the incidence and outcomes of NLA and describe the NLA services over 4 years from 1 January 2018 to 31 December 2021 in Bhutan.

## METHODS

### Study design, setting, and population

This retrospective study was conducted at Gyaltsuen Jetsun Pema Birthing Center, a facility within the JDWNRH. Data were collected from the records of women who received NLA between 1 January 2018 and 31 December 2021. The data were obtained from the Anesthesia Department, medical records section, admission forms, and birth‐registers. Women scheduled for elective cesarean section and who had received previous NLA during the study period were excluded.

### Study variables

The data collected were maternal age, parity, body mass index, education level, type of delivery (spontaneous, assisted delivery, and cesarean section), type of NLA (single‐shot spinal, CSE, and epidural), indication for NLA (labor pain, medical reason), newborn's birth‐weight, Apgar score at 1 and 5 min after birth, neonatal jaundice, NICU admission, and maternal complications from NLA. An Apgar score of less than 7 (0–6) is defined as a “low Apgar score,” and more than 7 (7–10) is defined as a “normal Apgar score.”

Data on NLA technique were also collected. These data were based on the records of the administration of NLA to patients. During this procedure, an informed written consent was obtained from the mothers. The NLA was provided under sterile conditions as per the department's standard operating procedure. Before the NLA was provided, the anesthesiologist and obstetrician screened mothers for medical and obstetric contraindications. The CSE technique was used if the mother was in severe pain. Otherwise, only epidural technique was used. However, if cervical dilatation was more than 7 cm, a single‐shot spinal analgesia was provided. The epidural catheter was inserted at L3/4 or L4/5. After confirming the epidural space with the loss of resistance technique, a 4‐ or 5‐cm catheter was kept in the epidural space. A test dose of 2–3 mL of 0.125% bupivacaine was administered before a full dose of analgesia was provided. After confirming the catheter was in the epidural space with a test dose, bupivacaine 0.125%–0.0625% with fentanyl 2 mcg/mL was administered via a patient‐controlled epidural analgesia (PCEA) machine. The nurses monitored mothers’ blood pressure, heart rate, pain score, and fetal heart rate every 5 min in the first 30 min and then every 30 min until the delivery for any abnormalities. After the third stage of labor was completed, the epidural catheter was removed.

### Data analysis

Data were recorded using Microsoft Excel version 16.57 (22011101) and analyzed using Epi Info 7.2.5.0. Descriptive statistics were used for the analysis. Categorical data were summarized using frequencies and percentages. Continuous data were first assessed for normal distribution. Those normally distributed were summarized using mean and standard deviation.

### Ethical considerations

The study was approved by the Research Ethics Board of Health of Bhutan (Ref No. REBH/2021/117) and cleared by the Medical Education and Research Unit (MERU) of the hospital (JDWNRH/MERU/01/2020‐2021/5084). The collected data are in the safe custody of the principal investigator, protected with a password. Throughout the data collection, mothers’ identity was kept confidential. Information from the client was obtained from the assigned registration number instead of the name.

## RESULTS

A total of 15,119 women were admitted for vaginal delivery at JDWNRH over a 4‐year period. Of that, 524 parturient mothers had received NLA. No woman had received more than one NLA during the study period. The incidence rate of NLA was 3.5%. The mean age of women was 27 ± 4.7 years (youngest 14 and eldest 44). More nulliparous mothers preferred NLA than the multiparous mothers (82.3% vs. 17.6%). Overall, 49.1% mothers had an education level up to the secondary level. By occupation, 48.5% of NLA recipients were homemakers. Details about maternal social demographic and characteristics of neonates born are in Table 1.

**TABLE 1:** Sociodemographic characteristics of women who received neuraxial analgesia for labor pain and childbirth in Bhutan, 2018–2021.

Characteristics	Frequency (*N* = 524)	%
Age of women when NLA received (years)		
Mean 27 ± 4.7, SE (0.21), 95% CI (26.56–27.37), age range (14–44)		
10–20	24	4.6
20–30	369	70.4
30–40	125	23.9
≥40	6	1.2
BMI (*N* = 484)		
≤18.5	1	0.2
18.6–24.9	98	20.3
25–29.9	262	54.1
≥30	123	25.4
Occupation (*N* = 517)		
Corporate	21	4.1
Government	155	30.0
Housewives	250	48.5
Private	87	16.9
Students	3	0.6
Education level (*N* = 515)		
Illiterate	44	8.5
Primary	26	5.1
Secondary	253	49.1
Tertiary	140	27.2
Graduate	47	9.1
Master	5	1.0
Indication for NLA (*N* = 524)		
Labor pain	490	93.5
Labor pain with medical condition	34	6.5
Technique of NLA (*N* = 523)		
Combined spinal epidural analgesia	262	50.1
Epidural analgesia	206	39.4
Single‐shot spinal analgesia	55	10.5
Mode of delivery (*N* = 523)		
Spontaneous vaginal delivery	331	62.4
Cesarean	96	18.4
Assisted vaginal delivery	96	18.4
Newborn (*N* = 515)		
Male	253	49.1
Female	262	50.9
Birth weight in grams (*N* = 506)		
<3000	129	25.5
>3000	377	74.5

Abbreviations: BMI, body mass index; NLA, neuraxial labor analgesia.

### Indication of neuraxial labor analgesia

NLA was administered to 93.5% (490/524) of pregnant mothers in labor pain (without medical conditions) and 6.4% (34/524) of pregnant mothers in labor pain (with medical conditions). Cardiac problems (64.71%) were the most common indication among medically indicated (Tables 2).

**TABLE 2:** Medical conditions of women who received neuraxial analgesia for labor pain and childbirth in Bhutan, 2018–2021.

Medical condition	Frequency (*N* = 34)	%
Anxiety	1	2.9
Epilepsy	2	5.9
Hypertension in pregnancy	8	23.5
Multiple comorbidities	1	2.9
Diabetic cardiomyopathy	1	2.9
Valvular heart disease	7	20.6
Rheumatic heart disease	12	35.3
Pulmonary hypertension	1	2.9
Arrhythmia (PSVT)	1	2.9

Abbreviation: PSVT, paroxysmal supraventricular tachycardia.

### Mode of delivery

The modes of delivery following NLA were spontaneous vaginal delivery, cesarean section, and assisted delivery—63.3%, 18.4%, and 18.4%, respectively. Failed progressions of labor (36.5%) and non‐reassuring fetal status (37.5%) were common indications for cesarean section (Table 3). Poor maternal effort (55.2%) was the commonest reason for assisted vaginal delivery (Table 4).

**TABLE 3:** Indication for cesarean section among women who received neuraxial analgesia for labor pain and childbirth in Bhutan, 2018–2021.

Characteristics	Frequency (*N* = 96)	%
Failed progress of labor	35	36.5
Non‐reassuring fetal status	36	37.5
Cephalopelvic disproportion	4	4.2
Malpresentation	5	5.2
Failed assisted vaginal delivery	4	4.2
Poor maternal effort	3	3.1
Missing data	9	9.4

**TABLE 4:** Indication for assisted vaginal delivery among women who received neuraxial analgesia for labor pain and childbirth in Bhutan, 2018–2021.

Characteristics	Frequency (*N* = 96)	%
Failed progress of labor	4	4.2
Non‐reassuring fetal status	28	29.2
Cardiac disease^a^	7	7.3
Poor maternal effort	53	55.2
Missing	4	4.2

^a^In cardiac disease, assisted vaginal delivery was conducted as prophylaxis.

### Technique of neuraxial labor analgesia

CSE analgesia, epidural analgesia, and single‐shot spinal analgesia were administered at 50.1%, 39.4%, and 10.5%, respectively. The CSE or epidural was preferred when cervical dilatation was less than 6 cm, and single‐shot spinal was preferred when cervical dilatation was more than 7 cm (Table 5). The loss of resistance with a loss of air technique, and PCEA was used in all CSE and epidural analgesia.

**TABLE 5:** Selection of neuraxial analgesia in relation to cervical dilatation among women who received neuraxial analgesia for labor pain and childbirth at tertiary Hospital, Bhutan in 2018–2021.

	Cervical dilatation	
Characteristics	0–6 cm^a^	7–8 cm^a^
Single‐shot spinal analgesia	14 (3.0)	41 (66.1)
Combined spinal epidural analgesia	245 (53.3)	16 (25.8)
Epidural analgesia	201 (43.7)	5 (8.1)
Total	460 (100)	62 (100)

^a^Values are number (%).

### Maternal complications

The maternal complications from NLA in our study were only seen in 1.5% of the participants (Table 6). No known major complications, such as cardiac arrest, death, high block, paraplegia, meningitis, and seizure, were reported.

**TABLE 6:** Neonatal outcome and maternal complications among women who received neuraxial analgesia for labor pain and childbirth in Bhutan, 2018–2021.

	Frequency (*N*)	%
Neonatal outcome		
Apgar score		
At 1 min (*N* = 511)		
0–6	26	5.1
7–10	485	94.9
At 5 min (*N* = 511)		
0–6	1	0.2
7–10	510	99.8
NICU admission (*N* = 524)		
Yes	4	0.8
No	520	99.2
Maternal complications		
Accidental dural puncture	5	1.0
Post‐dural puncture headache	1	0.2
Accidental removal of epidural catheter	1	0.2
Fever	1	0.2

Abbreviation: NICU: neonatal intensive care unit.

### Fetal complication

At 1 min, 26 neonates (5.1%) had Apgar scores less than 7 (0–6); at 5 min, only one neonate (1.2%) had a score less than 7. Four (0.8%) neonates were admitted to NICU due to respiratory depression, birth asphyxia, and meconium aspiration (Table 6). Neonatal jaundice was observed in 43 neonates (8%). No neonatal death following NLA was recorded.

## DISCUSSION

### Incidence rate of neuraxial labor analgesia

The incidence rate of NLA at the JDWNRH in Bhutan was 3.5%. The incidence was lower than that in the USA, France, China, and Japan [9, 10, 11, 12]. Nevertheless, those data are from the developed countries where labor analgesia existed for more than five decades [13]. However, the incidence rate of NLA in Bhutan is higher than 1.6% in Gauteng Province, South Africa [15]. Data on labor neuraxial or epidural analgesia from middle‐ and low‐income countries are limited for comparison. The shortage of anesthesia personnel, cost, cultural beliefs, lack of knowledge and availability of NLA, and scarcity of research may be the reason for the low rate of NLA.

### Age and previous pregnancy on deciding neuraxial analgesia

We found that those under the age of 30 and nulliparous received NLA more than those over 30 years and who were multiparous. A study in Lithuania found similar findings, with 26 years as the mean age of mothers receiving NLA [16]. We can speculate that younger women are less able to cope with pain and are more anxious than older women. For instance, in China, fear of labor pain and delivery was the number one maternal request for cesarean section [17]. Moreover, fear, non‐familiarity, and no experience coping with pain may also explain why nulliparous women opted for labor analgesia. A study using the MCGILL pain questionnaire found that primiparous women experienced a higher level of pain in the first stage of labor than multiparous women [1]. In contrast, a study in Vietnam reported that NLA was preferred more by women aged more than 35 years and those who were multiparous [18].

### Effect of neuraxial analgesia on delivery

With NLA, the rate of cesarean and assisted vaginal delivery was less than spontaneous vaginal delivery. This finding was consistent with previous studies [5, 6, 7, 16]. But, a Cochrane review of 23 trials found that NLA increased the rate of instrumental deliveries (RR 1.42, 95% CI 1.28–1.57) compared to non‐NLA [5]. However, confounding factors like nulliparous who visit hospitals earlier with a higher fetal station, slower cervical dilation, large babies, small pelvic outlet, and use of more potent analgesia might have biased the result [7]. Similarly, the concentration of local anesthetic is also reported to have an effect on the mode of delivery [19].

### Maternal complications

Five women had an accidental dural puncture during an epidural procedure, and only one reported the symptoms of post‐dural puncture headache. Therefore, the incidence was low similar to the findings of a study in the USA [20].

### Fetal complication

In our study, neonatal Apgar scores less than 7 at 1 and 5 min after birth were 26 (5.1%) and 1 (0.8%), respectively. Only four (0.8%) infants were admitted to NICU. The findings are consistent with previous randomized trials and systemic reviews [5, 7]. One study found neonates born from epidural exposure were 1.89 times (95% CI 1.45–2.46, *p*‐value <0.001) more likely to be admitted to NICU than those not exposed to epidural analgesia [8].

We observed neonatal jaundice in 8% (43) of the neonates. There is limited literature on neonatal jaundice with or without NLA. Studies in the 1970s and 1980s have postulated bupivacaine and cephalhematoma from instrumental vaginal delivery as an association with neonatal jaundice [21, 22]. However, recent studies did not show an association between bupivacaine and neonatal jaundice. Instead, the studies found delayed feeding as the cause [23, 24]. To find the association, we could not find data on neonatal cephalohematoma and women without epidural analgesia. Future studies can validate the association.

### Challenges and factors for low incidence

Multiple factors could have contributed to the low NLA rate in our study. First, the concept of NLA is new to Bhutanese women, which started only in 2016. A previous study found that only 61.4% of women knew about the availability of NLA services in the center [25]. Women and their attendants might be hesitant without local data on the safety of NLA. Future studies on the knowledge and attitude of NLA among laboring mothers, attendants, and health‐care staff might give a clear insight.

Another reason could be the country's limited number of anesthesiologists, midwives, and nurses. Women who receive neuraxial analgesia require close blood pressure monitoring, signs, and symptoms of local anesthesia toxicity, and continuous fetal cardiotocography monitoring. Although the service was available 24 × 7, it depended on the availability of the on‐call anesthesiologist. Similarly, when the patient load was high, midwives could not call anesthesiologists even when women demanded. The shortage of health workers in Japan and China has also led to a low incidence of labor epidural analgesia [11, 26], which corroborates with our setting. As of January 2022, Bhutan has only six national anesthesiologists (0.9 per 100,000); two are on study leave. The number and ratio of anesthesiologists to population are far below the minimum standard recommended by the World Federation Society of Anesthesiologist (WFSA): Five anesthesiologists per 100,000 people [27]. COVID‐19 has further worsened the shortage of health workers and health services, including labor epidural analgesia. Moreover, several lockdowns and COVID‐19 restrictions have made NLA inaccessible to other districts in the past 2 years, which corresponds to our study period. Researchers could explore the shortage of anesthesiologists and midwives in future studies to determine how it impacts NLA services and the quality of care it provides.

Culture, psychosocial, and attendant support also affect the decision to use labor epidural analgesia. Most Asian cultures believe birth is a natural process; pain builds the mother–child bond and represents the strength of the woman [11]. Due to these misconceptions, the NLA rate was low even in developed Asian countries like Japan and China [1, 26]. Those beliefs are also seen among Bhutanese. A study found that only 67% of Bhutanese women were willing to accept NLA [25]. In the same study, even after knowing the availability of NLA service, still, 23.5% refused NLA. In Saudi Arabia, fear of side effects such as backache and dural puncture was the main reason for not choosing labor epidural analgesia [28]. It could be the other reason preventing many Bhutanese mothers from opting for NLA.

Nearly half (48.94%) of our study population were homemakers. More than two thirds (76.42%) of these housewives have educational attainment up to the secondary level. In our study, the preference for NLA was less in those who had studied below the secondary level. Similar to our observation, in Vietnam, it has been observed that among mothers, the level of education had an influential role in their decision to opt for NLA [18]. In this study, graduates were four times more likely to use epidural analgesia than those educated up to high school [18].

Educating women during antenatal visits might help women and their partners to prepare mentally. In China, No Pain Labor and Delivery (NPLD) program educated mothers on the benefits of epidurals. The project was a success, with a 50% increase in epidural rate [17]. Similarly, studies found that educating mothers during antenatal visits increases women's preference for NLA [25, 28]. However, in Bhutan, no such advocacy or studies have been conducted. Collaboration between the Department of Anesthesiology and the Ministry of Health can promote epidural analgesia for mothers and conduct studies on its safety.

Our study was the first study on NLA in Bhutan. Therefore, it provides baseline information for future studies. However, this study has limitations. It is a retrospective descriptive study without comparison with the “no epidural group.” The available data are from routinely collected clinical records. Therefore, there are missing data such as the duration of labor (first and second stage), maternal hypotension, pruritus, urinary retention, failed NLA, breakthrough pain, and birth injuries to the mother (genital tract, cervical tear, and postpartum hemorrhage), and neonates (such as cephalohematoma, shoulder dystocia). In this study, no causal association was observed owing to its design.

## CONCLUSION

NLA has been established to be safe for both mother and fetus, but the number of women receiving NLA in Bhutan remains low. The scarcity of anesthesiologists and low awareness among mothers and health workers have hindered the delivery of service of labor analgesia. A collaborative effort between the Department of Anesthesia and the Ministry of Health can promote it.

## AUTHORS CONTRIBUTION

*Conceptualization; data curation; formal analysis; investigation; methodology; resources; software; supervision; validation; visualization; writing—original draft; writing—review and editing*: Tenzin Yoezer. *Conceptualization; formal analysis; investigation; methodology; resources; supervision; validation; visualization; writing—review and editing*: Dawa Gyeltshen, Jampel Tshering.

## CONFLICT OF INTEREST STATEMENT

Dr. Dawa Gyeltshen is an Editorial Board member of Public Health Challenges and a coauthor of this article. To minimize bias, he is excluded from all editorial decision‐making related to the acceptance of this article for publication.

## ETHICS STATEMENT

Not applicable.

## Data Availability

The data that support the findings of this study are available on request from the corresponding author. The data are not publicly available due to privacy or ethical restrictions.

## References

[1] Niven C , Gijsbers K . A study of labour pain using the McGill pain questionnaire. Soc Sci Med. 1984;19(12):1347‐1351. 10.1016/0277-9536(84)90023-6 6531713

[2] This Practice Bulletin was developed by the American College of Obstetricians and Gynecologists' Committee on Practice Bulletins—Obstetrics in collaboration with Lauren Plante, MD, MPH, and Robert Gaiser, MD . ACOG Practice Bulletin No. 209: obstetric analgesia and anesthesia. Obstet Gynecol. 2019;133(3):e208‐e225. Available at: https://www.zdrav.by/pdf/1167.pdf. 10.1097/AOG.0000000000003132 30801474

[3] World Health Organization . Intrapartum Care for a Positive Childbirth Experience. World Health Organization. 2018:212. 30070803

[4] Mathur VA , Morris T , Mcnamara K . Cultural conceptions of women's labor pain and labor pain management: a mixed‐method analysis. Soc Sci Med. 2020;261:113240. 10.1016/j.socscimed.2020.113240 32758799

[5] Anim‐Somuah M , Smyth RMd , Cyna AM , Cuthbert A . Epidural versus non‐epidural or no analgesia for pain management in labour. Cochrane Database Syst Rev. 2018;5(5):CD000331. 10.1002/14651858.CD000331.pub4. Published 2018 May 21. 29781504 PMC6494646

[6] Aragão FFDe , Aragão PWDe , Martins CA , Leal KFCS , Tobias AF . Neuraxial labor analgesia: a literature review. Braz J Anesthesiol (English Ed). 2019;69(3):291‐298. 10.1016/j.bjane.2018.12.014 PMC9391899 30777350

[7] Ashagrie HE , Fentie DY , Kassahun HG . A review article on epidural analgesia for labor pain management: a systematic review. Int J Surg Open. 2020;24:100‐104.

[8] Salameh KM , Anvar Paraparambil V , Sarfrazul A , Lina Hussain H , Sajid Thyvilayil S , Samer Mahmoud A . Effects of labor epidural analgesia on short term neonatal morbidity. Int J Womens Health. 2020;12:59‐70. 10.2147/IJWH.S228738. Published 2020 Feb 4. 32099485 PMC7007791

[9] Butwick AJ , Bentley J , Wong CA , Snowden JM , Sun E , Guo N . United States state‐level variation in the use of neuraxial analgesia during labor for pregnant women. JAMA Netw Open. 2018;1(8):e186567. 10.1001/jamanetworkopen.2018.6567. Published 2018 Dec 7. 30646335 PMC6324365

[10] Blondel B , Coulm B , Bonnet C , Goffinet F , Le Ray C . Results from the French National Perinatal Survey 2010: situation in 2016 and trends since 2010. J Gynecol Obstet Hum Reprod. 2017;46:669‐681. 29031048 10.1016/j.jogoh.2017.09.002

[11] Maeda Y , Takahashi K , Yamamoto K , Tanimoto T , Kami M , Crump A . Factors affecting the provision of analgesia during childbirth, Japan. Bull World Health Organ. 2019;97(9):631‐636. 10.2471/BLT.19.230128 31474776 PMC6705500

[12] Mu Yi , Wang X , Wang Y , et al. The trends and associated adverse maternal and perinatal outcomes of labour neuraxial analgesia among vaginal deliveries in China between 2012 and 2019: a real‐world observational evidence. BMC Med. 2021;19(1):74. 10.1186/s12916-021-01941-6. Published 2021 Mar 19. 33736635 PMC7977606

[13] Skowronski GA . Pain relief in childbirth: changing historical and feminist perspectives. Anaesth Intensive Care. 2015;43(suppl):25‐28. 10.1177/0310057X150430S106 26126073

[14] Jigme Dorji Wangchuck National Referral Hospital . Annual Report 2021 . Thimphu: Jigme Dorji Wangchuck National Referral Hospital. 2022.

[15] Leonard T , Perrie H , Scribante J , Chetty S . An audit of the labor epidural analgesia service at a regional hospital in Gauteng Province, South Africa. S Afr J Obstet Gynecol. 2018;24(2):52‐56. 10.7196/SAJOG.2018.v24i2.1314

[16] Rimaitis K , Klimenko O , Rimaitis M , Morkūnaitė A , Macas A . Labor epidural analgesia and the incidence of instrumental assisted delivery. Medicina (Kaunas). 2015;51(2):76‐80. 10.1016/j.medici.2015.02.002 25975875

[17] Hu LQ , Flood P , Li Y , et al. No pain labor & delivery: a Global Health Initiative's impact on clinical outcomes in China. Anesth Analg. 2016;122(6):1931‐1938. 10.1213/ANE.0000000000001328 27195636

[18] Nguyen LD , Nguyen AD , Farber MK , et al. Sociodemographic factors associated with request for labor epidural analgesia in a tertiary obstetric hospital in Vietnam. Biomed Res Int. 2021;2021:8843390. 10.1155/2021/8843390. Published 2021 Jan 30. 33604386 PMC7868158

[19] Sultan P , Murphy C , Halpern S , Carvalho B . The effect of low concentrations versus high concentrations of local anesthetics for labour analgesia on obstetric and anesthetic outcomes: a meta‐analysis. Can J Anaesth. 2013;60(9):840‐854. 10.1007/s12630-013-9981-z 23925722

[20] Delgado C , Bollag L , Van Cleve W . Neuraxial labor analgesia utilization, incidence of postdural puncture headache, and epidural blood patch placement for privately insured parturients in the United States (2008‐2015). Anesth Analg. 2020;131(3):850‐856. 10.1213/ANE.0000000000004561 31804407

[21] Wood B , Culley P , Roginski C , Powell J , Waterhouse J . Factors affecting neonatal jaundice. Arch Dis Child. 1979;54(2):111‐115. 10.1136/adc.54.2.111 434885 PMC1545363

[22] Clark DA , Landaw SA . Bupivacaine alters red blood cell properties: a possible explanation for neonatal jaundice associated with maternal anesthesia. Pediatr Res. 1985;19(4):341‐343. 10.1203/00006450-198519040-00004 4000759

[23] Ozcakir HT , Lacin S , Bay B , Luleci N , Sungurtekin Inceboz U . Segmental epidural anesthesia have no effect on neonatal serum bilirubin concentrations and neonatal jaundice. Turkiye Klinikleri J Gynecol Obstet. 2004;14:16‐20.

[24] Siyah Bilgin B , Altun Koroglu O , Yalaz M , Karaman S , Kultursay N . Factors affecting bilirubin levels during first 48 hours of life in healthy infants. Biomed Res Int. 2013;2013:316430. 10.1155/2013/316430 23841060 PMC3690209

[25] Tshomo T , Tenzin K , Tshering J . Awareness and perception of epidural labor analgesia among pregnant women visiting antenatal clinic in National Referral Hospital. NJOG. 2021;16(1):10‐15.

[26] Zhao P , Cai Z , Huang A , et al. Why is the labor epidural rate low and cesarean delivery rate high? A survey of Chinese perinatal care providers. PLoS One. 2021;16(5):e0251345. 10.1371/journal.pone.0251345. Published 2021 May 21. 34019570 PMC8139447

[27] Kempthorne P , Morriss WW , Mellin‐Olsen J , Gore‐Booth J . The WFSA Global Anesthesia Workforce Survey. Anesth Analg. 2017;125(3):981‐990. 10.1213/ANE.0000000000002258 28753173

[28] Alakeely MH , Almutari AK , Alhekail GA , et al. The effect of epidural education on primigravid women's decision to request epidural analgesia: a cross‐sectional study. BMC Pregnancy Childbirth. 2018;18(1):124. 10.1186/s12884-018-1766-5. Published 2018 May 3. 29724183 PMC5934814

